# Dynamics of Dilute Nanoalloy Catalysts

**DOI:** 10.1021/acs.jpclett.4c01659

**Published:** 2024-07-26

**Authors:** Rasmus Svensson, Henrik Grönbeck

**Affiliations:** Department of Physics and Competence Centre for Catalysis, Chalmers University of Technology, SE-412 96 Göteborg, Sweden

## Abstract

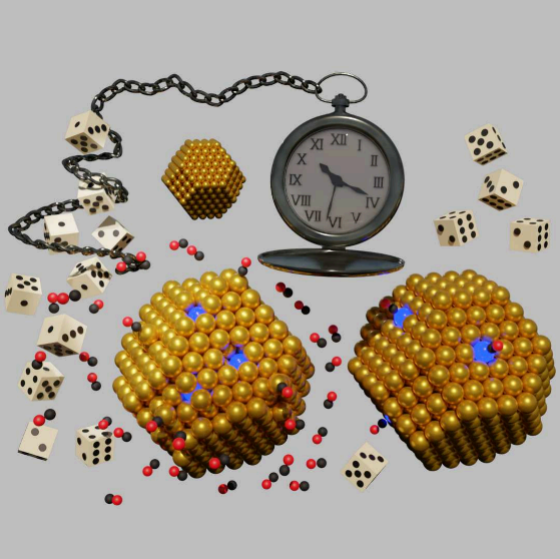

Capturing the dynamic character of metal nanoparticles under the reaction conditions is one of the major challenges within heterogeneous catalysis. The role of nanoparticle dynamics is particularly important for metal alloys as the surface composition responds sensitively to the gas environment. Here, a first-principles-based kinetic Monte Carlo method is developed to compare the dynamics of dilute PdAu alloy nanoparticles in inert and CO-rich atmospheres, corresponding to reaction conditions for catalyst deactivation and activation. CO influences the dynamics of the activation by facilitating the formation of vacancies and mobile Au-CO complexes, which are needed to obtain CO-stabilized Pd monomers on the surface. The structure of the catalyst and the location of the Pd monomers determine the rate of deactivation. The rate of catalyst deactivation is slow at low temperatures, which suggests that metastable structures determine the catalyst activity at typical operating conditions. The developed method is general and can be applied to a range of metal catalysts and reactions.

Heterogeneous catalysts are generally realized as metal nanoparticles (NPs) supported on porous oxides.^1^ The metal particles are dynamic at elevated temperatures and respond sensitively to the reaction conditions. Early measurements revealed that the shape of ZnO-supported Cu NPs changed with the composition of a H_2_/CO atmosphere.^2^ Adsorbates such as CO and NH_3_ can result in surface roughening^3^ and formation of small metal clusters and islands on extended transition-metal surfaces.^4^ CO has, moreover, been proposed to induce formation of single-atom Au catalysts by detachment of atoms from Au NPs supported on CeO_2_ during CO oxidation.^5^ Similarly, ligated Cu^+^ ions have been identified as intermediates in the reconstruction of copper electrocatalysts during CO_2_ reduction.^6^ The role of NP dynamics is particularly important for metal alloys, where the surface composition depends on the reaction conditions. The surface composition of RhPd particles has shown reversible changes when exposed to oxidizing and reducing conditions.^7^

The dynamics of metal alloy NPs becomes crucial in the dilute limit, where transition atom monomers are embedded in a noble metal host, so-called single-atom alloy (SAA) catalysts. SAA catalysts have shown to be highly selective for a range of reactions, including partial hydrogenation of hydrocarbons over PdCu^8,9^ and PdAu,^10−12^ hydrogenation of propenal over PdAg,^13^ and direct H_2_O_2_ formation from H_2_ and O_2_ over PdAu.^14,15^ The performance of these SAA catalysts is, however, conditional on the location of Pd monomers in the surface layer of the NP. Moreover, the activity and selectivity may depend sensitively on the coordination of the Pd atoms in the NP surface.^8,15^

Pd monomers dispersed in an Au host are in an inert atmosphere preferentially located in the bulk of the NP.^16^ This is a consequence of the stronger interaction between Au–Pd compared to the Au–Au interactions and the lower surface energy of Au. The Pd monomers in the surface could, however, be stabilized by adsorbates, such as CO.^16,17^ Thus, dilute PdAu NPs are commonly pretreated in a reactive atmosphere, such as CO and O_2_, to obtain active catalysts with Pd monomers in the NP surface.^18^

Capturing the dynamic character of metal NPs during reaction conditions is one of the major challenges within heterogeneous catalysis. Computationally, it is difficult as the metal dynamics generally require simulations times not accessible by molecular dynamics simulations.^19,20^ Here, we address this issue by developing a kinetic Monte Carlo (kMC) method with kinetic parameters obtained from density functional theory (DFT) calculations. The method is applied to the dynamics of dilute PdAu NPs focusing on catalyst activation in a CO atmosphere and deactivation in an inert atmosphere. The simulations account for surface and bulk processes and elucidate different roles of CO during NP activation. CO mediates the formation of vacancies, which facilitate metal subsurface diffusion and stabilize Pd monomers in the surface. The simulations reveal the governing mechanisms for the activation and deactivation of dilute PdAu and uncover the reversible formation of Au clusters on NP surfaces. The introduced computational procedure is general and can be used to address the dynamics of various metal NPs within heterogeneous catalysis.

DFT calculations are used to sample the potential energy landscapes for Pd monomers in Au(111), Au(100), and Au(211). The generated database describes the potential energy landscape over NPs. Access to the potential energy landscapes enables kinetic Monte Carlo simulations of NP dynamics during CO-promoted activation and inert-environment deactivation of dilute PdAu alloy NPs.

Potential energy landscapes are constructed by using either explicit calculations or scaling relations. Explicit values are used for CO desorption, a two-atom mechanism for vacancy formation/annihilation, diffusion by an exchange reaction, and metal (M) diffusion in the bulk or between surface layers. Scaling relations are used for a one-atom mechanism for vacancy formation/annihilation, CO-mediated vacancy formation/annihilation, and M and M-CO diffusion in/on the surface. The database is reported in the Supporting Information.

Having a database of DFT energies, we construct the complete potential energy landscapes for extended surfaces and nanoparticles. Figure 1 shows the landscape for Pd diffusion from the bulk to the Au(111) surface (catalyst activation). The formation of a vacancy and an adatom located in an fcc hollow site has in the absence of CO a barrier of 1.35 eV. The barrier for vacancy annihilation is very low, indicating that the vacancies in Au(111) are short-lived. However, the adatom can with a barrier of only 0.12 eV diffuse from the vacancy, which would increase the lifetime of the vacancy. The diffusion of Pd from the bulk to the subsurface layer is visualized with a dashed line. This step involves a series of vacancy-driven diffusion events. Pd diffusion in the bulk is enabled by the presence of neighboring vacancies. The endothermic diffusion of Pd to the surface has a barrier of 0.62 eV. Note that a vacancy is left in the second layer, which may after a series of diffusion events (dashed line) be filled with an Au atom. Creating Pd in the surface without CO is limited by three factors. (i) The formation of short-lived vacancies, which is associated with high barriers. (ii) Diffusion of Pd to the subsurface layer, which is a complex process conditional on neighboring vacancies. (iii) The activated system is endothermic with respect to Pd in the Au bulk.

**Figure 1:**
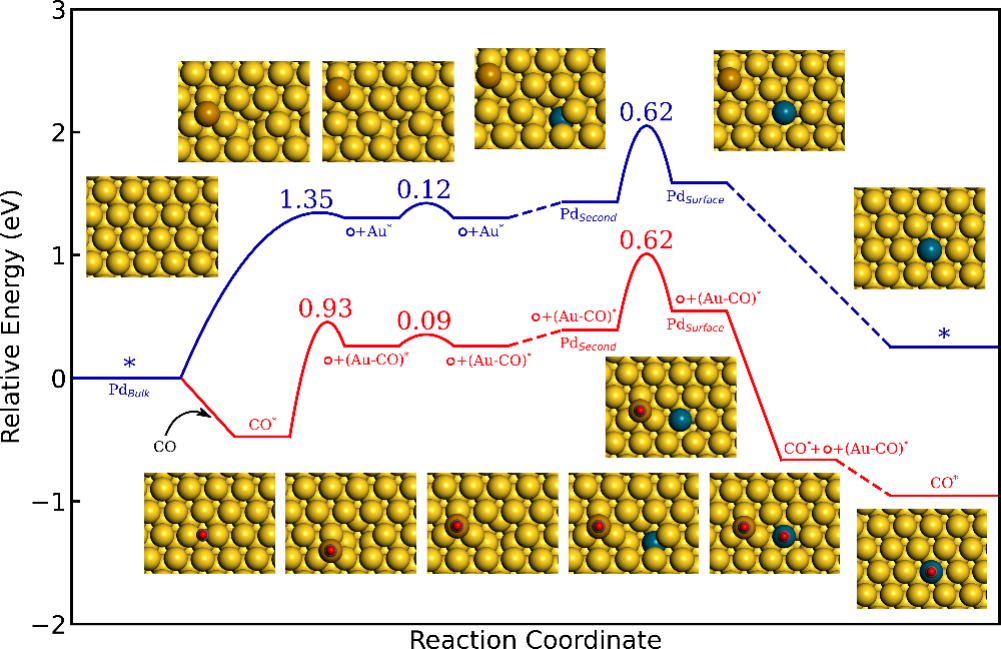
Potential energy landscape for the vacancy driven activation of Pd embedded in Au(111). Pd is initially located in the Au bulk. The red and blue paths are activation with and without CO, respectively. Species adsorbed on the surface are represented with *, whereas a vacancy is represented with . Atomic color codes: Au (yellow), Au adatom (brown), Pd (blue), C (gray), and O (red).

The situation is markedly different in the presence of CO. CO adsorbs bridging two Au atoms, with an adsorption energy of −0.48 eV. The formation of an Au-CO complex and a vacancy has a barrier of 0.93 eV. The barrier is lower than that without CO as the surface atom is solvated during the formation of the adatom. Importantly, CO stabilizes the Au adatom on the surface, making the barrier for vacancy annihilation higher than the diffusion barrier of the Au-CO complex away from the vacancy. The barriers for diffusion of Pd to the subsurface layer and surface layer are not affected by the presence of CO on the surface. The Au adatom may in a series of subsequent steps fill the remaining vacancy. Diffusing a Pd monomer from the bulk to the surface is exothermic in the presence of CO. The presence of CO influences the activation process in different ways. (i) The barriers to form the adatom/vacancy pair is reduced. (ii) The barrier for vacancy annihilation is larger than the barrier for Au-CO complex diffusion. (iii) Diffusion of Pd to the surface is facilitated by the presence of a larger amount of vacancies. (iv) The overall process is exothermic.

We used kinetic Monte Carlo simulations to explore the activation and deactivation processes of dilute PdAu alloys by studying the kinetics with and without CO as a function of temperature. The diffusion of Pd monomers is mediated by vacancy formation, which motivates us to first investigate the formation and annihilation of vacancies in Au-only systems.

Adatom/vacancy formation and diffusion are investigated for six systems. The low-index surfaces Au(111) and Au(100) are treated using 10-layer *p*(20 × 20) surfaces with periodic boundary conditions. The influence of under-coordinated sites is investigated using an Au(111) system with two (211) steps. Vacancies in NPs are investigated for truncated octahedra (TOh) with sizes 2.7, 3.6, and 4.5 nm, respectively. The system-dependent vacancy dynamics is compared in Figure 2 by showing time-averaged adatom coverages. The coverage is defined as the number of adatoms divided by the number of surface atoms in the unperturbed system.

**Figure 2:**
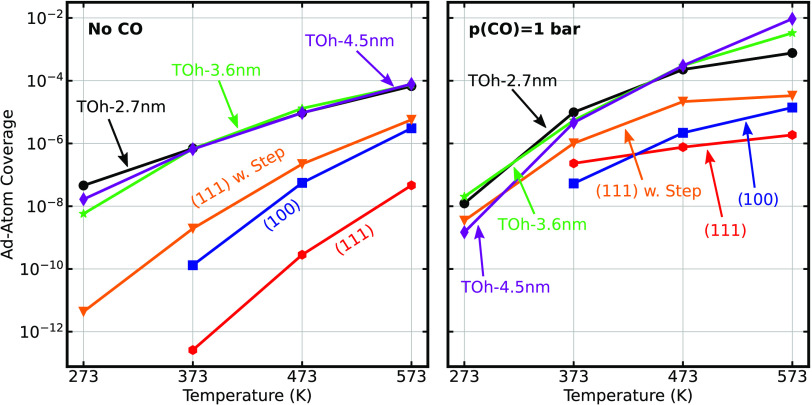
Coverage of adatoms as a function of temperature for Au(111) [red], Au(100) [blue], Au(111) with two (211) steps [orange], a 2.7 nm NP, [black], a 3.6 nm NP [green], and a 4.5 nm NP [purple]. Left: Inert atmosphere. Right: Atmosphere with 1 bar of CO pressure. The results are the averages of 16 independent kMC simulations.

The adatom coverage (and the number of vacancies) is in the absence of CO close to zero for Au(111) and Au(100) at 273 K. The coverages increase monotonically with temperature in the entire temperature interval; however, the coverage is still low for Au(111) at 573 K, being only 5 × 10^–8^. The presence of (211) steps substantially affects the formation of vacancies, being more than two orders of magnitude higher than for Au(111) in the entire temperature interval. The increased formation of adatom/vacancy pairs originate from the under-coordinated Au atoms at the step. The barrier to form a vacancy at the step is lower than to form a vacancy in the closed-packed surface. The adatom coverages are further increased for the nanoparticles. The dependence on particle size is small. The highest coverage is 8 × 10^–5^ at 573 K.

The presence of CO (1 bar) has clear effects on the formation of adatoms. The largest effect is observed for Au(111), which is a consequence of stabilization of the adatom. Vacancy formation in Au(111) without CO has an initial state with nine nearest neighbors, whereas the transition state has only two nearest neighbors. The high CO adsorption energy in the transition state decreases the barrier for the vacancy formation process. At the highest temperature (573 K), the adatom coverages are only slightly higher than those in the absence of CO, which is a consequence of a low CO coverage. The CO atmosphere slightly reduces the adatom coverage on the Au NPs at 273 K. The reduced adatom formation is related to a high CO coverage, which blocks sites for adatom formation and subsequent diffusion. At the intermediate temperatures (373 and 473 K), the adatom coverage is instead significantly increased for the Au NPs. The differences in adatom coverages between the different systems are reduced from ∼6 orders of magnitude without CO to only ∼2 orders of magnitude with CO. The reason is that the under-coordinated atoms, for which the vacancy formation process has low barriers in the absence of CO, are not as affected by the presence of CO as the higher-coordinated atoms.

Understanding the dynamic behavior of the vacancy formation is obtained by monitoring the time-evolution of the number of adatom/vacancy pairs. The number of adatoms as a function of time for three simulations of the 3.6 nm nanoparticle at a CO pressure of 1 bar at 573 K are shown in Figure 3. The simulations reveal that the formation of vacancies and the number of adatoms are autocatalytic. When an adatom/vacancy pair is formed, the coordination number of the Au atoms adjacent to the vacancy is decreased, lowering the energy barrier for the formation of a second adatom/vacancy pair. This is the reason for the common state having more than one adatom. The trajectories show cases with up to eight adatoms. These are cases in which the adatoms form clusters. The clusters are preferentially formed on the (100) facet, which is related to the higher stability of adatoms on the (100) facet (coordination number 4) compared to the (111) facet (coordination number 3). The coordination numbers increase upon clustering, which stabilizes the system. The lifetime of the clusters is generally below 1 μs. The adatom tetramer on the (100) facet is the core size from which larger clusters are formed. Once an adatom detaches from the tetramer cluster, the stability is reduced, and the cluster disintegrates.

**Figure 3:**
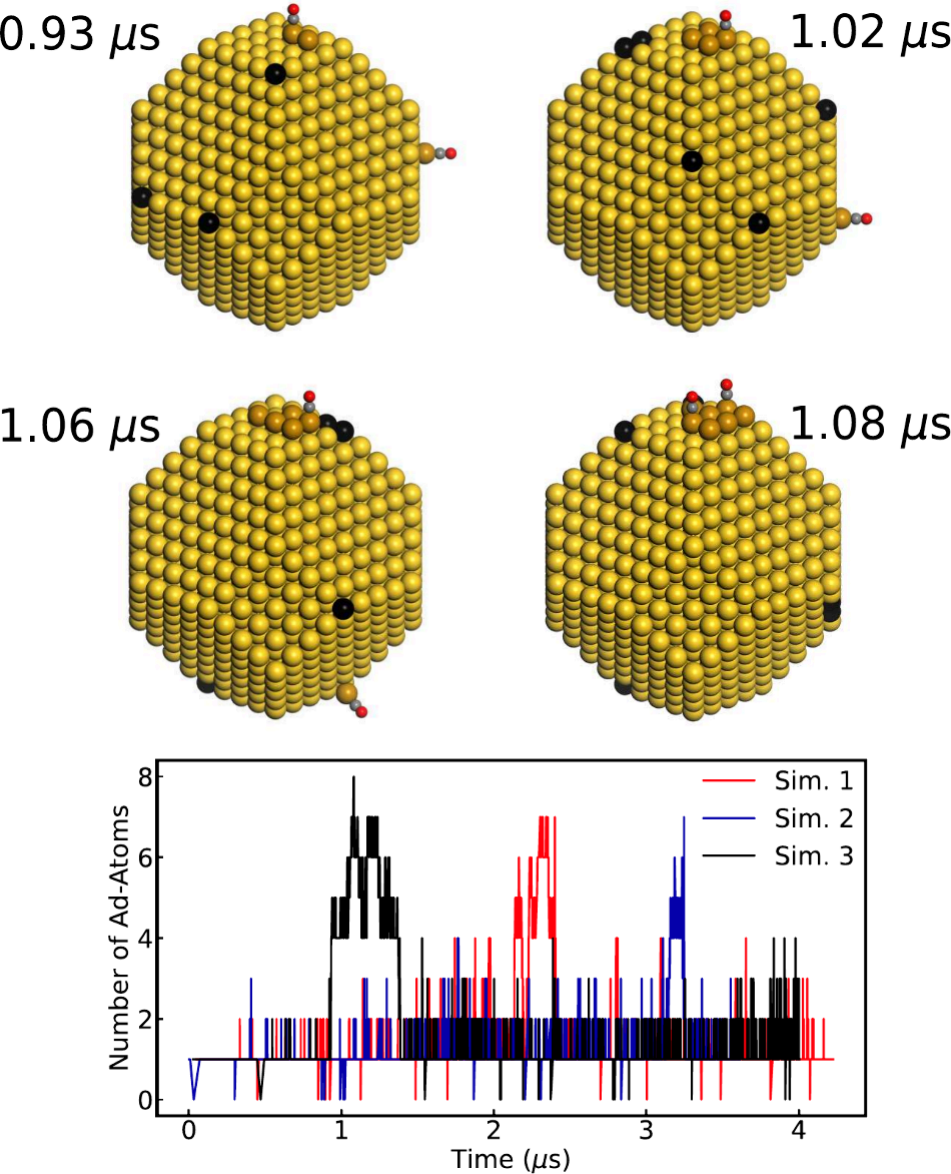
Top: Atomic models of snapshots along a trajectory of a 3.6 nm particle at 1 bar of CO pressure and 573 K. Atomic color codes: Au (yellow), Au adatom (brown), vacancy (black), C (gray), and O (red). Bottom: The number of adatoms for the 3.6 nm particle at a CO pressure of 1 bar at 573 K as a function of time for three independent simulations.

Snapshots along a trajectory exemplify the cluster formation. The starting configuration (not shown) is a bare unreconstructed nanoparticle. At 0.93 μs, three adatom/vacancy pairs have been formed, where two of the adatoms form a dimer. At subsequent times, the cluster grows to seven adatoms. It should be noted that single adatoms on the surface diffuse as Au-CO complexes. CO often desorbs upon clustering as the adsorption energy is decreased when the coordination number of Au is increased. Another reason for the low CO coverage on the clusters is the repulsion between the CO molecules. The vacancies (represented with black color) tend to form at the corners of the nanoparticle and also cluster, to minimize the surface energy of the particle.

The performance of dilute alloy NPs as selective catalysts^8,9,12−15^ is conditional on the presence of Pd in the surface, which is a metastable configuration in an inert atmosphere. Dilute PdAu systems are, therefore, commonly activated by pretreatment in a reactive atmosphere, such as CO.^18^

The activation of dilute PdAu systems involves a large number of events, of which the rates for CO adsorption/desorption are fast while the rates for vacancy formation is slow. To facilitate the exploration of the activation process in the presence of CO, we divided the kinetic Monte Carlo simulations into two parts. First, the probability of having a vacancy in the Au structure, *p*(vacancy) is obtained from Figure 2. Second, kinetic Monte Carlo simulations are performed with one vacancy (no adatom) in the system, obtaining a time for the Pd atom to reach the surface layer and adsorb CO (*t*_sim_). The time of activation (*t*_act_) is, thereafter, given by *t*_act_ = *t_sim_/p*(vacancy).

The Au(111) surface is investigated together with Au(111) with two (211) steps and a 3.6 nm TOh NP. The Pd monomer is for all systems initially placed in the subsurface layer of a (111) facet far from the vacancy. The activation times (*t*_act_) for the three systems are shown in Figure 4. The activation at 373 K is slow for all systems because of (i) the low number of vacancies, which is required for the Pd diffusion, and (ii) the slow rate of the subsurface Pd diffusion. The activation is particularly slow for the nanoparticle, as the vacancies are preferably located at the corners and edges of the NP, which limits the diffusion processes in the facets. The reason for the slow activation of the nanoparticle at 373 K as compared to the activation of Au(111) (with and without steps) is evident from Figure 2. The number of vacancies in the extended surfaces is only slightly lower than in the NP in the presence of CO; however, the distributions of the vacancies are different. The vacancies for the extended surfaces are relatively evenly distributed in the system, promoting the diffusion of Pd. Instead, the vacancies for the NP are locked to the corners, which does not facilitate Pd diffusion. The activation time is significantly decreased with increased temperature owing to both the increased number of vacancies and the faster diffusion of Au and Pd in the systems. The activation time is below 1 second at 573 K. However, it should be emphasized that the initial position of Pd in the systems may further influence the activation time.

**Figure 4:**
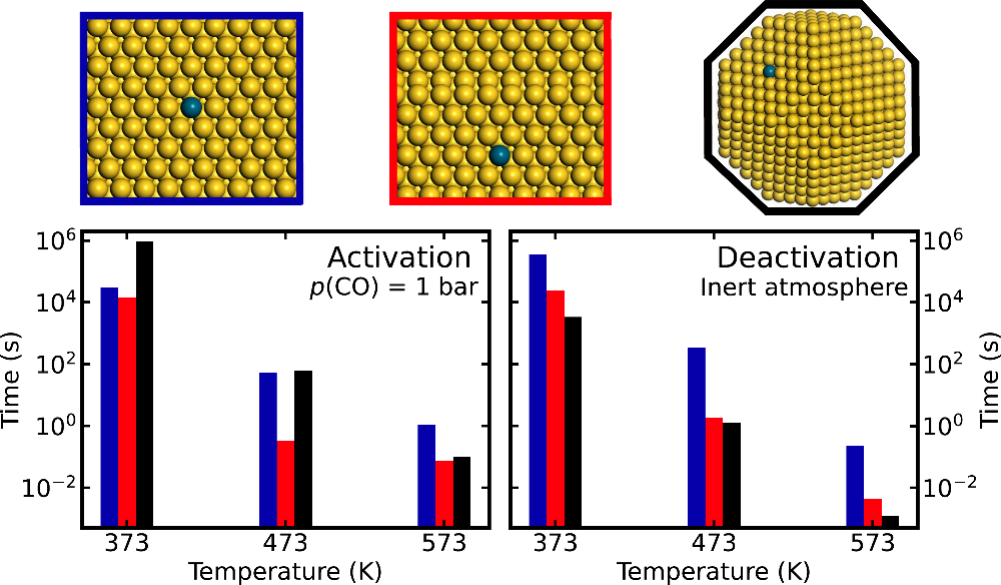
Top: Atomic models of the three explored systems, Au(111) [blue], Au(111) with two (211) steps [red], and a 3.6 nm nanoparticle [black]. Bottom left: The average activation time for when Pd was initially located in the (111) subsurface layer. Bottom right: The average deactivation time. The deactivation of Pd embedded in the Au(111) surface did not occur within 100 h at 373 K, and the bar is, therefore, set to 100 h. The activation and deactivation processes are reported for three temperatures, and the results are averages from several independent kinetic Monte Carlo simulations.

Having Pd monomers in the surface of a Au NP is a metastable configuration in the absence of a reactive gas atmosphere. Removing the reactive gas eventually results in deactivation of the dilute PdAu systems. The average time for deactivation for Pd embedded in the three systems is also shown in Figure 4. No deactivation is observed within 100 h for any of the systems at 273 K (not shown). Also at 373 K, no deactivation occurred within 100 h for a Pd monomer embedded in an extended Au(111) surface; thus, the bar is in this case set to 100 h. This result is in agreement with experiments showing that Pd monomers in dilute PdAu(111) alloys prepared at 380 K are predominantly located in the Au(111) surface.^21^ The system is at 473 and 573 K deactivated after ∼10 min and ∼0.2 s, respectively. The deactivation at higher temperatures agrees with experiments where Pd monomers are found to be located mainly in the subsurface layer upon deposition on Au(111) at 460 K.^22^

The rate of deactivation is significantly increased when steps are introduced to the extended Au(111) surface because of the increased number of vacancies in the system. However, the deactivation does not scale linearly with the number of under-coordinated atoms, as the vacancies are not evenly distributed (the vacancies are preferably located at under-coordinated positions). The deactivation is faster for all considered temperatures when Pd is embedded in an NP. The deactivation mechanisms are similar for all systems: Vacancies (preferably formed on under-coordinated positions) diffuse to the subsurface layer directly under the Pd monomer. The Pd monomer diffuses to the vacancy in the subsurface layer, and the vacancy diffuses away. Comparing deactivation and activation of the PdAu systems, we note that deactivation has a larger system dependence than activation, which is a consequence of the higher number of vacancies in the absence of CO for the NP. The slow kinetics at 373 K for the deactivation and activation suggests that metastable configurations of PdAu NPs can be present at typical temperatures for hydrogenation reactions.

The lifetime of Pd monomers in the Au surfaces of NPs depends on the specific location. Starting from different configurations, we estimate the lifetime of Pd monomers initially placed in a (111) facet, a (100) facet, an edge, and a corner of a 3.6 nm NP. The time evolutions of the position of the Pd monomers at 373 and 473 K for three randomly chosen simulations are shown in Figure 5. The Pd monomer is stable for ∼1 h at 373 K when placed in the (111) facet. The deactivation of the catalyst is much faster at 473 K, and the Pd monomer diffuses to the subsurface layer within ∼1 s.

**Figure 5:**
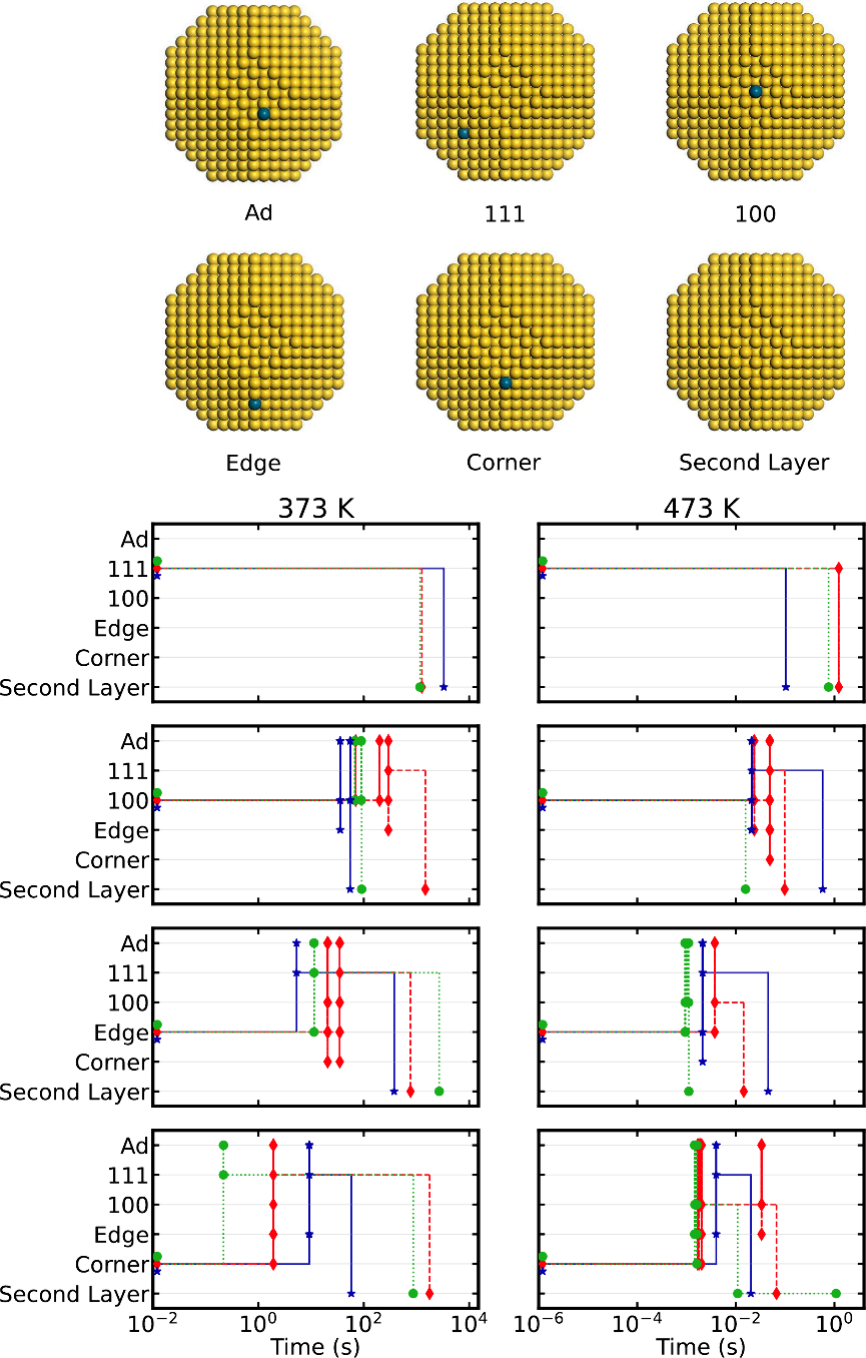
Position of the Pd monomer, obtained from kinetic Monte Carlo simulations, as a function of time at 373 and 473 K. In the top row, Pd is initially embedded in a (111) facet of the Au nanoparticle. In the second, third, and fourth rows, the Pd monomer is initially placed in a (100) facet, an edge, and a corner, respectively. Note the different time scales for the two temperatures.

For Pd initially placed in the (100) facet, the time for the deactivation differs between the different simulations at both 373 and 473 K. The spread in deactivation times is owing to the competing mechanisms in the deactivation process. Au adatoms located in the vicinity of the Pd monomer may at 373 K induce Pd adatoms via an exchange mechanism. The Pd adatom may, thereafter, diffuse over the surface or exchange position with an Au-atom located in the surface or heal a vacancy. The exchange mechanism is preferred over simply healing a vacancy as it does not require a vacancy close to the adatom. The deactivation is facile in cases where the Pd monomer is located in the (100) facet or at an edge. The mechanism for the deactivation of Pd in Au(100) is similar to the deactivation of Au(111). The lifetime of Pd-monomers initially placed in the (100) facets is extended if Pd first exchanges position with an Au adatom and thereafter diffuses to the (111) facet where it takes a position in the surface layer.

The situation when Pd is placed initially in an edge or at a corner is similar to when Pd is placed in a (100)-facet. If the Pd monomers are located in an under-coordinated position, the deactivation is facile. However, the facile deactivation competes with the diffusion of Pd adatoms to the (111) facet, where the deactivation is slower. The results for the deactivation show that the metastable configurations with Pd monomers in the surface layer are not instantly deactivated at 373 K, as the process is kinetically hindered. The time for deactivation is strongly dependent on the position of the Pd monomers. We do not observe deactivation at 273 K within 100 h for any of the initial positions of the Pd monomers.

The structure of metal NP catalysts depends sensitively on the operating conditions. We have developed a first-principles-based kinetic Monte Carlo approach to describe CO-enhanced dynamics of dilute PdAu alloys with Pd embedded in a Au host. The activity and selectivity of dilute PdAu alloys require Pd to be present in the NP surface. Pd is in an inert atmosphere preferentially located in the Au bulk, whereas the presence of CO stabilizes Pd in the Au surface. CO influences the dynamics of the activation by facilitating the formation of vacancies and mobile Au-CO complexes, which are needed to obtain CO-stabilized Pd monomers in the surface. The deactivation of the catalyst in the absence of CO depends on the position of the Pd monomer and the global structure of the catalyst. The extended lifetime of the systems with Pd in the surface at common reaction conditions underlines the robustness of the systems as catalysts for various hydrogenation reactions. The outlined approach to study the dynamics of nanoparticles in reactive environments is general and can be applied to a range of systems and reactions.

## Computational Methods

DFT calculations are performed using the Vienna Ab Initio Simulation Package (VASP).^23−26^ The frozen-core projector augmented-wave method^27,28^ is employed to describe the interactions between the core and valence electrons. The considered valence electrons are 2*s*^2^ 2*p*^2^ (C), 2*s*^2^ 2*p*^4^ (O), 5*s*^0^ 4*d*^10^ (Pd), and 6*s*^1^ 5*d*^10^ (Au). The exchange-correlation functional is approximated by the Perdew, Burke, and Ernzerhof (PBE) formula^29^ together with the Grimme-D3 correction,^30,31^ to capture van der Waals interactions. A cutoff energy of 450 eV is used to expand the Kohn–Sham orbitals. The electronic structure is considered to be converged when the change in electronic energy and Kohn–Sham eigenvalues between two succeeding iterations are below 1.0 × 10^–6^ eV. The atomic structures are optimized and the structures are considered to be converged when the largest forces on the nuclei are below 0.03 eV/Å.

To model the potential energy landscape of the nanoparticles, explicit calculations are performed for a Pd atom embedded in *p*(3 × 3) model surfaces of Au(111), Au(100), and Au(211). The model surfaces are constructed with six atomic layers, separated by 14 Å of vacuum. The bottom two layers are kept fixed to their bulk positions. The Au bulk lattice constant is used for all highly diluted systems.^32^ Pd is considered in the topmost layer, the second layer, and the third layer. The Brillouin zone is sampled with a Γ-centered (7,7,1) k-point mesh. The potential energy landscape for Pd in the bulk is evaluated from calculations in a (3,3,2) cell consisting of 72 atoms. The Brillouin zone is in this case sampled with a Γ-centered (3,3,5) k-point mesh. The lattice constant of bulk Au is calculated to be 4.10 Å, which is in good agreement with the experimental results of 4.08 Å.^33^

The energy barriers of the vacancy formation processes and the diffusion of Au and Pd atoms are determined using the Climbing Image Nudged Elastic Band (CI-NEB) method.^34,35^ The energy of gas-phase CO is calculated using a (30, 31, 32) Å box. The Brillouin zone is, in this case, sampled with only the Γ-point. Vibrational energy modes are determined assuming the harmonic approximation, calculated using the finite differences approach.

The barriers for CO-mediated metal-diffusion events are investigated with constrained *ab initio* Molecular Dynamics (AIMD) simulations. The model surfaces are in these cases constructed by four atomic layers, with the bottom two layers fixed to their bulk positions. The electronic structure is in the AIMD simulations considered converged when the change in electronic energy and Kohn–Sham eigenvalues between two succeeding iterations are below 1.0 × 10^–5^ eV. The time-step is set to 1 fs, and a temperature of 373 K is maintained using a Nosé-Hoover thermostat.

The dynamics of the nanoparticles are explored using kinetic Monte Carlo (kMC) simulations. kMC is a common stochastic approach to obtain the time evolution of adsorbates on a catalyst surface.^36,37^ Here, the kMC simulations are expanded to describe also the structural dynamics of the surface and the bulk of the catalysts by allowing for vacancy formation and annihilation, as well as (CO-mediated) metal diffusion. The considered elementary reactions are
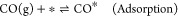
R1
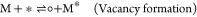
R2
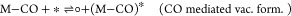
R3
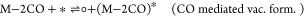
R4
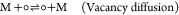
R5
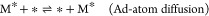
R6
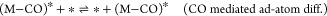
R7
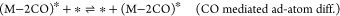
R8
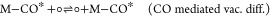
R9
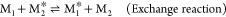
R10M denotes either Au or Pd,  denotes a vacancy anywhere in the metal system,  denotes an empty adsorption site, and * denotes a specie adsorbed on the surface (CO or an adatom). R1 describes CO adsorption and desorption. Reactions R2–R4 describe the formation or annihilation of a vacancy in the surface layer. In the vacancy formation, a metal atom (with or without an adsorbed CO molecule) moves to an empty surface site, forming an adatom on the neighboring surface site, and a vacancy. In the reverse reaction, the adatom moves to the vacancy in the surface. Reaction R5 describes the diffusion of a metal atom (on the surface or in the bulk), which is a process that depends on the presence of a neighboring vacancy in the structure. Reactions R6–R8 are diffusion of adatoms (with one or two CO molecules) on the metal surface. Reaction R9 describes the diffusion of an M-CO unit where M is part of the surface layer. The diffusion events with two CO molecules (R4 and R8) are relevant mainly for the Pd atoms. Elementary reaction R10 is a special diffusion event, describing an exchange reaction where an adatom diffuses to a surface position, whereas an atom originally in the surface forms an adatom; see Figure S1 in the Supporting Information.

The kinetic Monte Carlo simulations are performed using the First Reaction Method^38^ in which a list of time of occurrences is created for all possible elementary reactions. For each reaction that transfers the system from state *i* to state *j*, the time of occurrence *t*_*j*,*i*_^occ^ is given by
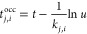
1where *t* is the current time in the simulation, *k*_*j*,*i*_ is the rate constant of the elementary reaction, and *u* is a uniform random number in the interval (0,1). The system is updated according to the elementary reaction with the shortest time of occurrence. In the update, the time of the system evolved and the structure of the catalyst changed according to the elementary reaction. The elementary reactions that are disabled after the update are removed from the list, whereas enabled events, with the corresponding times of occurrence, are added to the list.

Transition-state theory is used to calculate the rate constants used in the kinetic Monte Carlo simulation:
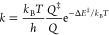
2where *Q*^‡^ is the partition function of the transition state (excluding the reaction coordinate), *Q* is the partition function of the initial state, and *ΔE*^‡^ is the activation energy of the elementary reaction. The prefactors for metal diffusion events are set to *k*_B_*T*/*h*. Thermodynamic consistency is ensured by calculating the rate constants for the reverse reactions from the equilibrium constants (*K*):
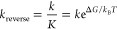
3where *ΔG* is the change in Gibbs free energy for the reaction.

## Supporting Information Available

Detailed description of the considered elementary reactions together with DFT calculations and scaling relations (PDF)
