# Supplementary material for: Dynamics of Dilute Nanoalloy Catalysts
Source: J Phys Chem Lett. 2024 Jul 26;15(31):7885–91. doi: 10.1021/acs.jpclett.4c01659 (PMC11318031; doi:10.1021/acs.jpclett.4c01659)
Supplement: Supplementary file 1 — jz4c01659_si_001.pdf [file jz4c01659_si_001.pdf]

Supporting Information: Dynamics of Dilute Nanoalloy Catalysts

Rasmus Svensson* and Henrik Grönbeck*

Department of Physics and Competence Centre for Catalysis, Chalmers University of Technology, SE-412 96 Göteborg, Sweden

E-mail: rassve@chalmers.se; ghj@chalmers.se

DFT-generated potential energy landscapes

Potential energy landscapes are constructed using either explicit calculations or scaling relations. Explicit values are used for CO desorption, a 2-atom mechanism for vacancy formation/annihilation, diffusion by an exchange reaction and metal (M) diffusion in the bulk or between surface layers. Scaling relations are used for a 1-atom mechanism for vacancy formation/annihilation, CO-mediated vacancy formation/annihilation, and M as well as M-CO diffusion in/on the surface. Table S1 summarizes how the different adsorption and activation energies are calculated. The values for the different events are given in the following sections.

Table S1: An overview on how adsorption and activation energies are obtained from the DFT-calculations for each type of the reaction events.

	**Event**	**Reaction**	**Description**
1	CO adsorption/desorption	$CO(g) + * \rightleftharpoons CO^*$	Explicitly for each CN
2	Ad-atom diffusion	$M^* + * \rightleftharpoons * + M^*$	Scaling Relations
3	Ad-atom diffusion by exchange reaction	$M_1 + {M_2}^* \rightleftharpoons {M_1}^* + M_2$	Explicit calculations (On extended surfaces)<br>Scaling Relations (Between different CN)
4	CO-mediated ad-atom diffusion	$(M{-}xCO)^* + * \rightleftharpoons * + (M{-}xCO)^*$	Scaling Relations
5	Vacancy formation	$M + * \rightleftharpoons \circ + M^*$	Scaling relations (1-atom mechanism)<br>Explicit calculations (2-atom mechanism)
6	CO-mediated vacancy formation	$M{-}xCO + * \rightleftharpoons \circ + (M{-}xCO)^*$	Scaling relations
7	Vacancy diffusion	$M + \circ \rightleftharpoons \circ + M$	Explicit calculations (On extended surfaces)<br>Scaling Relations (Between different CN)
8	CO-mediated vacancy diffusion	$M{-}CO^* + \circ \rightleftharpoons \circ + M{-}CO^*$	Scaling Relations

1. CO adsorption/desorption

The adsorption energy of CO on Au is calculated explicitly on sites with different coordination numbers. The non-zero point corrected adsorption energies are given in Table S2. The preferred adsorption mode depends on the Au-coordination number. CO is adsorbed bridge for high coordination numbers and atop for low coordination numbers. The adsorption energy of a second CO molecules on asymmetric low-coordinated Au (≤ 5). The coordination number of the atop site is used in the kMC simulations in cases where CO is adsorbed in bridge mode. In the kMC simulations, the zero-point correction and the partition functions of CO adsorbed on Au are calculated from the atop site and bridge sites calculated on Au(111).

Table S2: Non zero-point corrected adsorption energies of CO and a second CO molecule on Au atoms with different coordination numbers in different structures. The coordination number (CN) is presented for the atop adsorption site.

Structure	Adsorption site	CN	E_{ads}(CO)	$E_{\mathrm{ads,\ second}}$(CO)
111	bridge	9	-0.531	-
100	bridge	8	-0.815	-
211	bridge	7	-0.849	-
532	atop	6	-0.928	-
1Ad@(211-m100)	atop	5	-1.023	-0.374
1Ad@(100)	atop	4	-1.022	-0.429
1Ad@(111-fcc)	atop	3	-1.141	-0.452
1Ad@(111-Bridge-Vac)	atop	2	-1.226	-0.505

CO adsorbs preferably on an atop position on the Pd monomers. A second CO molecule may adsorb on Pd monomers. In this case, both CO molecules tend to adopt positions between atop and bridge adsorption sites. In Table S3, the non-zero point corrected adsorption energies of one CO molecule and two CO molecules on Pd are presented. In the kMC simulations, the zero-point correction and the partition functions of CO adsorbed on the Pd monomers are calculated from the vibrational modes obtained from adsorption on a Pd monomer embedded in Au(111).

Table S3: Non zero-point corrected adsorption energies of CO and a second CO molecule on Pd monomers with different coordination numbers in different structures. Note that upon adsorption of a second CO molecule, the adsorption sites changes towards top/bridge sites.

Structure	Adsorption site	CN	E_{ads}(1 CO)	$E_{\text{ads, second}}$(CO)
10fold (211 step)	bridge	10	-1.170	-0.108
111	atop	9	-1.274	-0.615
100	atop	8	-1.468	-1.071
211	atop	7	-1.480	-1.160
532	atop	6	-1.572	-1.155
1Ad@(211-m100)	atop	5	-1.583	-1.071
1Ad@(100)	atop	4	-1.464	-1.268
1Ad@(111-fcc)	atop	3	-1.685	-1.196
1Ad@(111-Bridge)	atop	2	-1.687	-1.287

The pairwise CO-CO adsorbate-adsorbate interaction is explicitly calculated for Au(111) to be 0.14 eV. This interaction is used for all CO-CO interactions on the surface, or between CO molecules adsorbed on neighboring ad-atoms.

2. Ad-atom diffusion

The diffusion energy barrier is calculated as the difference between the energy of the transition state and the initial state configuration:

$$E_{\text{a}} = E_{\text{ads,TS}}(\text{M}) - E_{\text{ads,initial}}(\text{M}), \tag{1}$$

The energies of the initial state and the transition state are calculated with respect to the gas phase metal (M) atom according to:

$$E_{\text{ads}}(\text{M}) = E_{\text{structure+M}} - E_{\text{structure}} - E_{\text{gas}}(\text{M}), \tag{2}$$

The adsorption energy/stability of an Au or Pd atom at different positions in or on the surface is determined by the use of scaling relations, based on the coordination number. The scaling relation is obtained by calculating the energy of Au and Pd for different positions over a range

of model surfaces. The results are shown in Table S4. Explicit calculations on Au(111) reveal that the formation of islands stabilizes ad-atoms. The energy of the transition state to or from islands is unaffected by the neighbors of the initial and final states. Hence, the diffusion barrier for ad-atoms approaching an island is unaffected, whereas the diffusion away from a neighboring ad-atom on the surface is increased. The explicitly calculated CO adsorption

Table S4: The adsorption energy of Au/Pd in or on different surfaces. In the first seven rows, a metal atom is filling a vacancy in the extended surfaces. In the last 10 rows, the metal atom resides on the surface as an ad-atom.

Structure	CN	E_{ads}(Au)	E_{ads}(Pd)
111	9	-4.287	-5.043
100	8	-4.198	-4.687
211 (step)	7	-3.968	-4.465
532	7	-3.957	-4.451
511	7	-4.199	-4.696
335	7	-3.939	-4.798
532	6	-3.699	-4.162
Ad-532	6	-3.687	-4.154
Ad-211	5	-3.477	-3.869
Ad-532	5	-3.473	-3.842
Ad-511	5	-3.230	-3.829
Ad-335	5	-3.480	-3.883
Ad-100 Hollow	4	-3.292	-3.638
Ad-111 FCC	3	-2.955	-3.198
Ad-211	3	-2.869	-3.103
Ad-532	3	-2.851	-3.041
Ad-111 Bridge	2	-2.836	-3.112

energies and metal adsorption energies are presented in Figure S1. The relative stability of ad-atoms and surface atoms (Au or Pd) scales linearly with the coordination number of the considered atom, top left in Figure S1. The larger slope for a Pd atom explains why Pd preferably occupy subsurface positions in an inert atmosphere. The adsorption energy of CO on Au and Pd sites on the nanoparticle do not follow the same linear trend, and are therefore described explicitly as a function of CN in the kMC simulations.

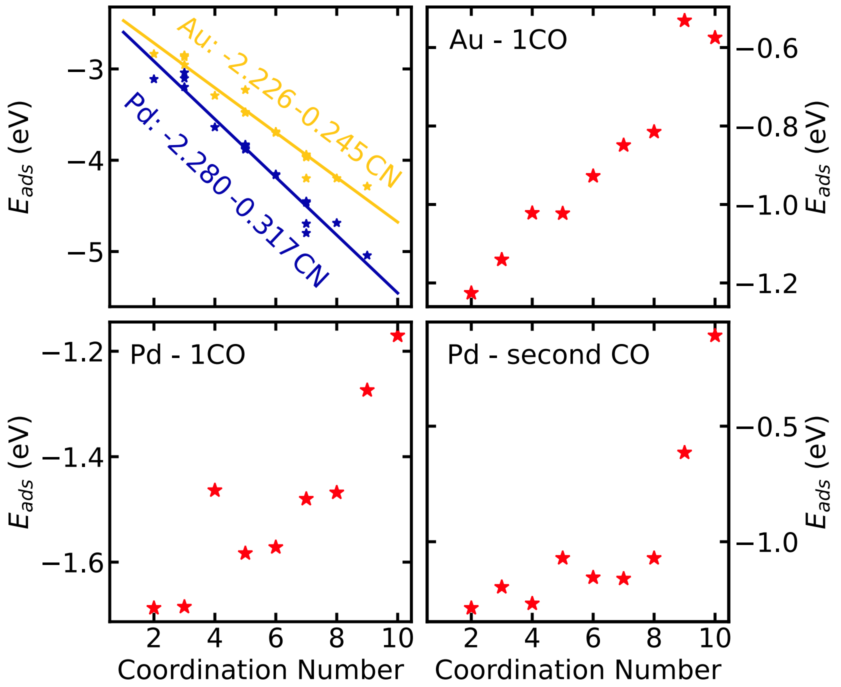

Figure S1: Top left: Adsorption energy of an Au atom (yellow) and a Pd atom (blue) with respect to the corresponding gas-phase atoms. Top right: The adsorption energy of CO over Au with different coordination numbers. Bottom left: The adsorption energy of CO on Pd with different coordination numbers. Bottom right: The adsorption energy of a second CO on Pd with different coordination numbers. Scaling relations are used to describe metal stability, whereas explicit values are used for CO adsorption energy.

3. Ad-atom diffusion by exchange reaction

A special case of ad-atom diffusion is the interchange between an ad-atom and a surface atom (R10 in the main text), visualized in Figure S2. The exchange event, where an ad-atom diffuses to a surface position whereas an atom originally in the surface forms an ad-atom, may occur via 2 Au atoms, or 1 Au atom and 1 Pd atom. The activation energy of the exchange mechanism for the different sites of the nanoparticles are obtained from the explicitly calculated energies (Table S5) scaled with the coordination of the ad-atoms and the number of neighboring Au/Pd ad-atoms in the initial state, according to the scaling relations in in Figure S1. Interestingly, the exchange mechanism (two Au atoms) is kinetically favorable over the normal surface diffusion on Au(100) and on Au(211). It should also be

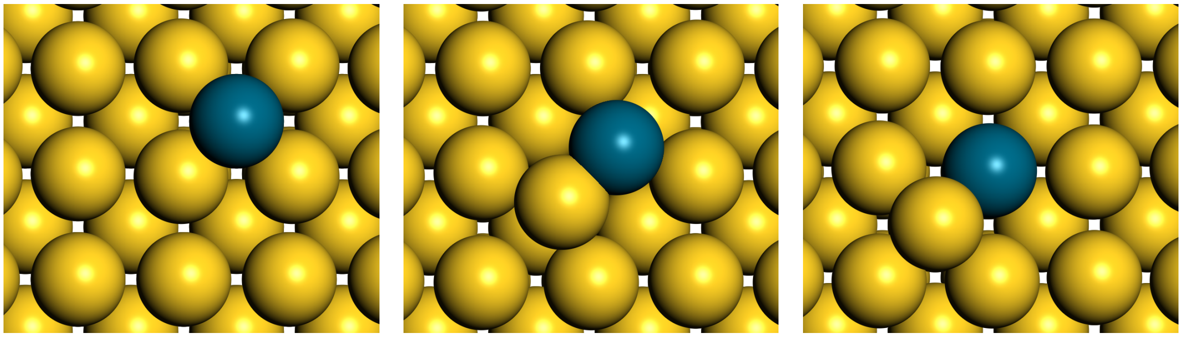

Figure S2: Atomic ball-model of the exchange reaction R10 in the main text. A Pd ad-atom replaces an Au surface atom while an Au ad-atom is formed. Atomic color codes: Au (yellow) and Pd (blue).

noticed that the exchange mechanism, in which Pd forms an ad-atom has a lower activation energy than the 1-atom and 2-atom vacancy creation mechanisms with Pd. Hence, the Pd ad-atoms formed in the deactivation simulations originate from the exchange mechanism. The exchange mechanism is also the main route for Pd ad-atoms going to the surface layer, even though the barrier is lower for the vacancy annihilation processes. This is a consequence of the kinetics in the simulations, as the exchange mechanism do not require a vacancy in the surface to occur.

Table S5: Non zero-point corrected energy barriers for the exchange mechanism, where an ad-atom takes a surface position, whereas the surface atom forms an ad-atom. In the first column, the extended surface is presented. In the second and third columns, the initial and final states of the reaction are presented. In column 4 and 5, the forward and backward reaction barriers are presented. When assigning the states, the first atom M(ad) represents the ad-atom, whereas M(surf) represents the surface atom (M: Au or Pd). For processes involving only Au, the initial and final states of the exchange process are similar to a normal surface diffusion, therefore, the barrier for normal surface diffusion is included in the last column for comparison. Figure S2 shows the backward reaction for the (100) surface (fifth structure).

Structure	Initial State	Final State	E_a forward [eV]	E_a backwards [eV]	E_a(Normal Diffusion) [eV]
111	Au(ad) + Au(surf)	Au(surf) + Au(ad)	0.876	0.876	0.119
100	Au(ad) + Au(surf)	Au(surf) + Au(ad)	0.342	0.342	0.515
211	Au-3fold(ad) + Au(step)	Au(step) + Au-5fold(ad)	0.166	0.774	0.250/0.8581
111	Au(ad) + Pd(surf)	Au(surf) + Pd(ad)	1.548	0.915	-
100	Au(ad) + Pd(surf)	Au(surf) + Pd(ad)	0.713	0.462	-
211	Au-3fold(ad) + Pd(step)	Au(step) + Pd-5fold(ad)	0.384	0.837	-
211	Au-5fold(ad) + Pd(step)	Au(step) + Pd-3fold(ad)	1.0831	0.145	-

4. CO-mediated ad-atom diffusion

The activation energy for the diffusion of Au-CO and Pd-CO complexes are calculated as:

$$E_{\mathrm{a}} = E_{\mathrm{TS}}(\mathrm{M}-\mathrm{CO}) - E_{\mathrm{ini}}(\mathrm{M}-\mathrm{CO}). \tag{3}$$

The stability of the transition state and the initial state configurations are calculated using the scaling relations of the metal-adsorption energy, and the explicitly calculated adsorption energy of CO, as a function of CN (Figure S1). Hence, the stability is constituted of two energy contributions according to:

$$E(\mathrm{M}-\mathrm{CO}) = E_{\mathrm{ads}}(\mathrm{M}) + E_{\mathrm{ads}}(\mathrm{CO}). \tag{4}$$

In the case of neighboring M-CO complexes, the diffusion barrier in decreased by 0.14 eV, as the energy of the initial state is increased by the CO-CO repulsion. Note that for both Au and Au-CO ad-atom diffusion, the initial state is stabilized by neighboring Pd atoms, according to the scaling relations (0.317 - 0.245) eV, hence increasing the energy barrier of

the diffusion.

5. Vacancy formation energy

Two mechanisms are considered for vacancy formation, *i.e.* when a surface atom leaves the original surface position to form an ad-atom/vacancy pair. In the first mechanism (one-atom creation/annihilation), the surface atom diffuses from the surface to form an ad-atom, in an otherwise unchanged surface. In the second mechanism, two-atom creation/annihilation, a neighboring surface atom follows the atom diffusing to the surface, taking its place in the surface. This mechanism increases the coordination of the diffusing atom in the vacancy formation process. However, the diffusion of the neighboring surface atom is also associated with a small activation energy.

The vacancy formation/annihilation energy barrier is in the 1-atom mechanism found to follow the scaling relations obtained for the metal adsorption energies, *i.e.*,

$$E_{\mathrm{a}} = E_{\mathrm{ads,TS}}(\mathrm{M}) - E_{\mathrm{ads,ini}}(\mathrm{M}), \tag{5}$$

where E_{ads} are obtained from scaling relations. The activation energies of the 1-atom vacancy formation/annihilation mechanisms are, hence, described in the same way as ad-atom diffusion.

The activation energy of the 2-atom vacancy formation/annihilation mechanism does not follow linear scaling relations. Therefore, the activation energies are obtained by explicit calculations. The activation energy of the 2-atom vacancy creation is lowered with a constant shift of 0.07 eV and 0.20 eV in the presence of neighboring vacancies on Au(111) and Au(100), respectively. The shift for Au(111) is also used for the under-coordinated Au sites. Note that the annihilation barrier both for the 1-atom and 2-atom mechanism is increased in the case of neighboring ad-atoms on the surface, according to the scaling relations presented in Figure S1.

In Table S6, explicitly calculated energy barriers for the vacancy formation and annihilation processes involving one and two Au atoms, respectively, are shown for the two mechanisms. In Table S7, explicitly calculated energy barriers for the vacancy formation and annihilation processes involving one Pd atom - and 1 Au atom and one Pd atom, respectively, are shown for the two mechanisms. Note that the two-atom vacancy formation mechanisms are associated with considerably lower barriers for atoms with high coordination numbers, whereas the energy barriers are similar for lower coordination numbers. The same trend is observed for the annihilation of the vacancies. Vacancies formed via the one-atom mechanism may be annihilated via the two-atom mechanism, and vice versa. In the kMC simulations, the mechanism with the lowest energy barrier for each site is implemented.

Table S6: Non zero-point corrected energy barriers for Au vacancy creations and annihilation for the 1-atom and 2-atom mechanisms.

Structure	CN	1-atom creation [eV]	1-atom annihilation [eV]	2-atom creation [eV]	2-atom annihilation [eV]
111	9	1.570	0.237	1.346	0.043
100	8	1.457	0.525	0.992	0.060
211 to m(100)	7	0.805	0.115	0.930	0.389
211 to 111	7	1.310	0.242	1.228	0.133
335	7	1.255	0.143	1.176	0.127
511	7	1.185	0.487	0.970	0.221
EDGE (modified 101)	7	1.274	0.259	1.216	0.200
532	6	0.892	0.073	1.009	0.175

Table S7: Non zero-point corrected energy barriers for Pd vacancy creations and annihilation for the 1-atom and 2-atom mechanisms.

Structure	CN	1-atom creation [eV]	1-atom annihilation [eV]	2-atom creation [eV]	2-atom annihilation [eV]
111	9	2.320	0.449	1.965	0.093
100	8	1.917	0.849	1.840	0.813
211 to m(100)	7	1.023	0.112	1.086	0.437
211 to 111	7	1.861	0.506	-	-
335	7	1.778	0.326	1.433	0.094
511	7	1.218	0.376	1.191	0.310
EDGE (modified 101)	7	1.834	1.150	1.488	0.886
532	6	1.052	0.025	-	-

6. CO mediated vacancy formation energy

The CO mediated formation of vacancies (Au and Pd) are investigated with constrained AIMD simulations for Au(111) and Au(211). The AIMD simulations indicate that, in the presence of CO, the 1-atom mechanism is preferred. Thus, the scaling relations for Au/Pd atoms and the explicitly sampled CO adsorption energies are used to determine the energy barrier for these events, *i.e.*,

$$E_{\mathrm{a}} = E_{\mathrm{TS}}(\mathrm{M-CO}) - E_{\mathrm{ini}}(\mathrm{M-CO}), \tag{6}$$

where

$$E(\mathrm{M-CO}) = E_{\mathrm{ads}}(\mathrm{M}) + E_{\mathrm{ads}}(\mathrm{CO}). \tag{7}$$

7. Vacancy diffusion energies

The energies of metal atoms with higher coordination number, such as metal atoms resided in the sub-surface layers or in the bulk, do not follow the linear trend with respect to coordination number. The reaction energy and activation energy of Au and Pd diffusion in and between Au surface layers are therefore calculated explicitly for Au(111), Au(100), and Au(211) extended surfaces. The results are presented in Table S8 for Au diffusion and in Table S9 for Pd diffusion. In the kMC simulations of the nanoparticles, the diffusion between the second and third layers are chosen to be the value calculated for Au(111). The diffusion barriers within the third layer and below are chosen to be the same as the barriers in the bulk.

At the surface, the scaling relations for the energies of the initial and final configurations for Au and Pd hold. Hence, the activation energies are altered in the presence of vacancies (and in the presence of Pd for Au diffusion). The diffusion between different sites in the surface (facets, edges and corners) are assigned rates following a Brønsted-Evans-Polanyi relation. The activation energy is obtained from the sum of the diffusion in the Au(111)

surface, and a contribution which is half the relative stability between the final and initial states of the diffusion, *i.e.*,

$$E_{\mathrm{a}} = E_{\mathrm{a},(111)} + \frac{E_{\mathrm{ads,fin}}(\mathrm{M}) - E_{\mathrm{ads,ini}}(\mathrm{M})}{2}. \tag{8}$$

The vacancies are preferably dispersed in the bulk, which is opposite to the preference on the surface. The energy preference for dispersed vacancies over neighboring vacancies is 0.11 eV. Hence, the diffusion barrier from a higher coordinated position, to a lower coordinated position in the bulk is lowered by 0.11 eV, whereas the reverse reaction is unaffected. The interaction between Au and Pd in the bulk follows the scaling relations in Figure S1. (The activation energy for Au diffusion away from a Pd atom in the bulk is increased with (0.317 - 0.245) eV.)

Table S8: Non zero-point corrected energy barriers for the diffusion of Au in Au. In the first column, the surface is presented. In the second and third columns, the initial positions of Au and the vacancy are presented, respectively. In the fourth and fifth columns, the forward and backward energy barriers are presented. Note that the backward barrier is for the event when the second and third columns are interchanged.

Surface	Initial Layer	Final Layer	E_a forward [eV]	E_a backwards [eV]
111	1	1	0.360	0.360
111	1	2	0.398	0.244
111	2	2	0.341	0.341
111	2	3	0.302	0.371
100	1	1	0.373	0.373
100	1	2	0.188	0.178
100	2	2	0.347	0.347
100	2	3	0.247	0.468
211	1	1	0.389	0.389
211	1	2	0.097	0.293
211	2	2	0.335	0.335
211	2	3	0.200	0.402
Bulk	-	-	0.418	0.418

Table S9: Non zero-point corrected energy barriers for the diffusion of Pd in Au. In the first column, the surface is presented. In the second and third columns, the initial positions of Pd and the vacancy are presented, respectively. In the fourth and fifth columns, the forward and backward energy barriers are presented. Note that the backward barrier is for the event when the second and third columns are interchanged.

Surface	Initial Layer	Final Layer	E_a forward	E_a backwards
111	1	1	0.600	0.600
111	1	2	0.465	0.620
111	2	2	0.654	0.654
111	2	3	0.612	0.793
100	1	1	0.544	0.544
100	1	2	0.173	0.655
100	2	2	0.687	0.687
100	2	3	0.6093	0.845
211	1	1	0.569	0.569
211	1	2	0.100	0.818
211	2	2	0.645	0.645
211	2	3	0.554	0.788
Bulk	-	-	0.827	0.827

8. CO-mediated vacancy diffusion energies

A special kind of surface diffusion event is the diffusion of atoms in the surface, with an adsorbed CO molecule. Such events are relatively rate. Explicit AIMD simulations are performed for Au-CO and Pd-CO diffusion in the surface of Au(111) and reveal that the influence of CO is small on Au diffusion (activation energy 0.30eV), whereas the CO mediated diffusion process of Pd-CO is associated with a barrier of 0.37 eV. The energy barrier for the diffusion between different sites are shifted according the the relative stability of Au-CO and Pd-CO in the different surface sites, similar to the case of vacancy diffusions. The activation energy is calculated as:

$$E_{\mathrm{a}} = E_{\mathrm{a},(111)} + \frac{E_{\mathrm{fin}}(\mathrm{M}-\mathrm{CO}) - E_{\mathrm{ini}}(\mathrm{M}-\mathrm{CO})}{2} \tag{9}$$

$$= E_{\mathrm{a},(111)} + \frac{\big(E_{\mathrm{ads,fin}}(\mathrm{M}) + E_{\mathrm{ads,fin}}(\mathrm{CO})\big) - \big(E_{\mathrm{ads,ini}}(\mathrm{M}) + E_{\mathrm{ads,ini}}(\mathrm{CO})\big)}{2}. \tag{10}$$

For the diffusion of Pd-2CO in the surface, the barrier obtained from Pd-CO diffusion is used. However, the diffusion between different sites are altered, as the relative stability of

Pd-2CO differs from Pd-CO.

Adsorbate-adsorbate interactions

For all metal diffusion events (with or without CO), the Au-Pd contribution and CO-CO interactions are added after the Brønsted-Evans-Polanyi relations. The activation energy for Au diffusing away from Pd is affected by (0.317 - 0.245) eV, whereas the barrier for Au diffusing towards Pd is unaffected. Similarly, the barrier for an Au-CO diffusion event is lowered in the case of neighboring Au-CO complexes in the initial state. The diffusion towards other Au-CO complexes are unaffected. Similarly, the diffusion of Au/Pd towards neighboring vacancies has a higher activation energy 0.245 eV/0.317 eV.
